# Planning for a Healthy Aging Program to Reduce Sedentary Behavior: Perceptions among Diverse Older Adults

**DOI:** 10.3390/ijerph19106068

**Published:** 2022-05-17

**Authors:** Efekona Nuwere, Bethany Barone Gibbs, Pamela E. Toto, Sharon E. Taverno Ross

**Affiliations:** 1 Department of Occupational Therapy, Long Island University, 1 University Plaza, Brooklyn, NY 11201, USA; 2 Department of Health and Human Development, University of Pittsburgh, 4200 Fifth Avenue, Pittsburgh, PA 15260, USA; bbarone@pitt.edu (B.B.G.); seross@pitt.edu (S.E.T.R.); 3 Department of Occupational Therapy, University of Pittsburgh, 4200 Fifth Avenue, Pittsburgh, PA 15260, USA; pet3@pitt.edu

**Keywords:** sedentary behavior, sitting, older adults, healthy aging, qualitative, perceptions, program development, senior center

## Abstract

Reducing prolonged engagement in sedentary behavior is increasingly considered a viable pathway to older-adult health and continued functional ability. Community-based programs that aim to increase physical activity can improve programs’ acceptability by integrating older adults’ perspectives on sedentary behavior and healthy aging into their design. The purpose of this study was to better understand the perceptions of a diverse group of community-dwelling older adults regarding sedentary behavior and its influence on healthy aging. Six focus group discussions with forty-six participants took place across two senior centers in New York City. Self-report questionnaires about daily activity patterns, general health status, and typical sedentary behaviors were also completed by the participants and analyzed using descriptive statistics. The focus group discussions were audio-recorded, transcribed, and analyzed using inductive and deductive approaches and an ecological framework to identify salient themes. A qualitative analysis revealed that the participants were aware of the physical costs of engaging in prolonged sedentary behavior. However, many routine sedentary activities were perceived to be health-promoting and of psychological, cognitive, or social value. The insights gained can inform the development of senior-center programs and health-promotion messaging strategies that aim to reduce older adults’ sedentary behavior.

## 1. Introduction

Older adults are the least physically active [1,2] and most sedentary of all age groups [3,4,5], with a higher prevalence observed among women and minority populations [1,2,4]. Observational data suggest that prolonged sedentary behavior is independently associated with poor health among older adults [6,7]. Prolonged sedentary behavior has been linked to an increased risk of adverse health outcomes, such as metabolic syndrome, increased waist circumference, obesity, cardiovascular disease, and all-cause mortality [8,9,10,11,12]. The deleterious effects of excessive sitting are especially pronounced in aging populations as the cardiovascular and musculoskeletal systems weaken and social relationships change, increasing the risk of falls [13], frailty [14], and mobility impairment [15,16]. Owen et al. [17] present an ecological model of sedentary behavior that helps conceptualize the determinants of older adult sedentary behavior. Understanding these personal, social, and environmental influences gives a necessary context to patterns of sedentary behavior that can aid the development of policy interventions, messaging strategies, and healthy aging programs designed to help older adults better manage their sedentary time. Spending less time on prolonged sedentary activities may be an important protective factor in maintaining physical health and psychosocial wellbeing in late adulthood.

Previous qualitative studies [18,19,20,21,22,23,24,25] have explored older adults’ perceptions of sedentary behavior. The participants in these studies shared the ways in which excessive sitting affected their physical, cognitive, and psychosocial health. While these qualitative studies have contributed to our understanding of contextualized information about the individual and environmental factors influencing older adults’ sedentary behavior, they were conducted primarily in homogeneous (i.e., white, higher SES) samples. Such sampling biases limit our understanding of the perceptions of sedentary behavior from adults of socioculturally heterogeneous backgrounds and hamper the design of effective interventions for these communities. Few studies exploring older adults’ perceptions of sedentary behavior have included adults from diverse socioeconomic [23] or minority backgrounds [25]. In the study published by Warren et al. [25], culture-specific and broader societal factors were identified as contributors to the sedentary behavior patterns of a sample of African-American women. However, the majority of these participants were middle-aged (53.6 ± 6.0) and employed. A separate study with older adults from both low and high socioeconomic backgrounds [23] found that differences in community resources and access to local recreational activities were perceived to encourage a more socially isolated, sedentary lifestyle among the group of participants who lived in disinvested neighborhoods. While the inclusion of a socioeconomically diverse group captured a broader range of perspectives on sedentary behavior, the authors noted that the racial and ethnic homogeneity (primarily white British) of their sample limited the generalizability of their findings.

The published qualitative sedentary behavior studies designed to investigate sedentary behavior are largely limited to homogenous groups. It is unclear if these findings are consistent or relevant to more racially, ethnically, and socioeconomically heterogeneous groups. Our understanding of health behavior promotion among various populations is incomplete without adequate consideration of the socioeconomic and cultural dimensions of health [26,27]. Therefore, the present study applies a socioecological framework to explore perceptions of sedentary behavior and healthy aging among community-dwelling older adults from racially, ethnically, and socioeconomically diverse backgrounds in New York City.

## 2. Materials and Methods

### 2.1. Study Design and Setting

A needs assessment was conducted to gain a better understanding of the beliefs held about sedentary behavior among a group of multi-ethnic/racial and socioeconomically diverse older adults. Focus groups allowed the researchers to explore in depth the personal, sociocultural, and environmental realities of the participants’ daily activity patterns. The researchers took the necessary step of gathering older adults’ perspectives and input in an effort to co-create an acceptable healthy aging program that would have value and meaning for the participants [28,29]. Theories of ecological systems [17,30,31,32] and a framework for healthy aging [33,34,35] provided the conceptual foundation for this inquiry.

This needs assessment was conducted at two senior centers that had established academic-community partnerships through an academic program, located in New York City, that supported training (fieldwork) for occupational therapy students. One center is centrally located, within a large low-income public housing development in Queens, NY. The other is located on a commercial corridor in a middle- and upper-income neighborhood in Brooklyn, NY. Both centers are located in mixed (residential and commercial) areas rich in neighborhood amenities (e.g., parks, shopping, and public transportation).

### 2.2. Participants and Sample Selection

The sample consisted of older adults who attended one of the two aforementioned senior centers. Consistent with the inclusion criteria, participants were 60 years or older, showed a willingness to participate in a focus group interview, demonstrated an understanding of the aims and procedures of the study, and had sufficient ability to speak, write, and read English. Given the demographic and historical context of the neighborhoods in which the senior centers were situated, it was projected that the study participants would be racially, ethnically, and socioeconomically heterogeneous.

Researchers implemented a proactive, face-to-face recruitment plan that sought to mitigate potential recruitment barriers to focus group participation [36]. Participants were recruited over several sessions in January 2020 using a convenience sampling method.

Before going on-site to recruit, researchers met with the program directors to discuss the proposed study, conceptual framework, and anticipated outcomes. Once on-site, senior center staff and program directors facilitated introductions to potential participants, extending the circle of trust to the researchers. Additionally, senior-center personnel assisted with the posting of recruitment flyers and allowed the researchers to make recruitment announcements following the morning and lunch meetings. Recruitment flyers, consent forms, and an interpreter were available for Spanish-speaking participants.

### 2.3. Instrumentation and Data Collection

Six focus group interviews were conducted between January and March 2020. Focus group interviews lasted approximately 60 min and were moderated by the principal investigator in a semi-private area within each senior center shortly after lunchtime. At the beginning of each focus group interview, permission to audio-record was obtained verbally from all participants. After each focus group, participants completed a brief demographic questionnaire. As encouraged by Miles, Huberman, and Saldana (2014) [37], the principal investigator jotted debriefing notes of general observations and group dynamics at the end of each focus group interview to ensure initial ideas worthy of future consideration were documented and retrievable during data analysis. Participants were not compensated for their study participation, but light refreshments were provided during the focus groups. All study procedures and protocols were approved by the University of Pittsburgh Institutional Review Board (STUDY19070144).

Focus Groups. A semi-structured discussion guide was developed based on interview questions and findings from previously published qualitative studies on older adult sedentary behavior [18,19,20,21,22,23,24,25] and was used to facilitate focus group discussions. As recommended by Mertens (2015) [38], open-ended questions were asked to create dialogue and encourage participants to offer their perspectives on the development of a healthy aging program at the senior center. A definition of what was meant by the term sedentary behavior [39] was provided to participants at the beginning of each focus group, followed by specific examples of seated activities to help further elucidate the concept. Discussion questions prompted older adult participants to share their views on sedentary behavior and aging, the personal and environmental factors they perceived contributed to and prevented them from being sedentary, and which senior-center programs they recommended to help them become less sedentary. Focus group interviews were conducted until thematic saturation was reached and authors were reasonably assured no new themes would emerge from the data [40].

Descriptive Statistics. Socio-demographic (i.e., age, gender, race/ethnicity, educational attainment, employment status, relationship status, and living arrangements), health- and activity-related, and center utilization variables were collected to describe study participants. Activity-related items were adapted from two validated sedentary behavior measures [41,42] and the various sedentary activities were arranged into four ecological domains (leisure, transportation, work, and household). Additionally, participants estimated the average amount of time they spent sitting over the course of a day using a single-item visual analog scale [43].

### 2.4. Data Analysis

Microsoft Excel was used to perform descriptive statistics on the socio-demographic and health- and activity-related variables. AI-powered transcription software (Otter.ai, Los Altos, CA, USA) transcribed the audio-recorded focus group interviews, and the principal investigator verified the accuracy and corrected any transcription errors. ATLAS.ti (version 8.4.4, Scientific Software Development GmbH, Berlin, Germany) was used to store, organize, and manage the data retrieved from the interview transcripts.

An ecological framework provided the conceptual foundation for this study and informed the analysis and interpretation of data through personal, social, and environmental/organizational themes. Thematic analysis of the transcripts and jottings was conducted using a framework method for health research [44]. Specifically, the principal investigator independently coded each interview transcript using inductive and deductive thematic approaches. Inductive codes were generated through an iterative process to ensure important perspectives were not missed. Deductive codes were based on common themes reported in previous qualitative sedentary behavior studies. Codes and initial analyses were reviewed by a co-author (SETR) and any discrepancies were discussed to finalize the codebook.

## 3. Results

### 3.1. Demographic Characteristics

Forty-six (*n =* 46) older adults participated in six focus groups across two senior centers. The sample characteristics of all participants obtained by the center are available in Table 1. The mean age was 75.6 ± 7.8 years, with 89.1% of the participants being female. The majority of the participants (64.4%) identified as Black, Caribbean, African-American, or of Hispanic descent. About half (48.9%) of the participants reported obtaining a college degree or higher. Additionally, the majority of the participants reported being retired (89.67%), lived alone (60.86%), and visited the center a few times a week (45.6%) or daily (37.0%).

### 3.2. Health Status and Patterns of Daily Activity

The self-reported general health status and activity patterns of all the participants are shown in Table 2. The majority of the participants reported their general physical health as good (43.5%) or very good/excellent (45.7%). Similarly, most of the participants rated their overall mental health as either good (28.3%) or very good/excellent (58.1%). Most of the participants perceived themselves to be more physically active than their peers (68.9%) and reported that they regularly walked or biked to perform errands (82.61%). A majority of the participants reported that they engaged in at least 10 min of vigorous-intensity recreational activity, moderate-intensity recreational activity, and heavy household work most days of the week (75.0%, 60.6%, and 56.5%, respectively). With regards to the average time spent on sedentary behavior, participants reported they spent, on average, 4.5 ± 2.0 h sitting per day.

### 3.3. Older Adult Sedentary Behavior

**Figure 1:**
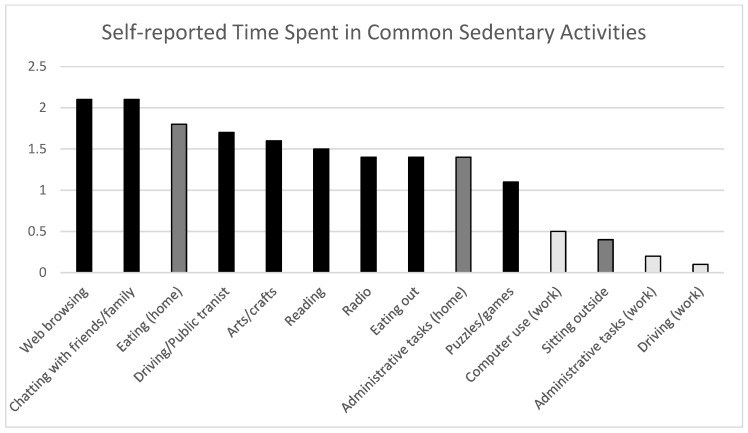
Participants’ self-reported time spent in various sedentary activities.

Figure 1 presents the results of the self-reported time spent on common sedentary activities within the leisure, work, transportation, and household domains. Overall, the participants reported spending most of their time on activities in the leisure domain and the least amount of time in the work domain. The top leisure-time activities were watching TV (2.6 h/day), going online (2.1 h/day), and sitting/talking with friends (2.1 h/day). Within the transportation domain, the participants, on average, reported spending 1.7 h/day sitting while using public transit or driving. The participants also reported the approximate time spent on household sedentary activities, such as having meals at home (1.8 h/day) and completing administrative tasks (e.g., bill-paying, making appointments) (1.4 h/day). Lastly, although this was infrequent, the participants reported that most of their time spent in the work domain involved computer use (0.5 h/day).

### 3.4. Focus Groups Results

The qualitative analysis yielded three main themes regarding the older adults’ views on sedentary behavior and healthy aging: (1) Avoiding sedentary behavior, enjoying seated activities; (2) multi-level influences on sedentary behavior; and (3) self-determining healthy aging. The themes, core categories, and sub-categories are presented in Table 3 and described with illustrative quotes below.

#### 3.4.1. Theme 1. Avoiding Sedentary Behavior, Enjoying Seated Activities

This theme revealed that the participants maintained a holistic view of sedentary behavior. On one hand, the participants attested to the adverse short-term health effects they felt as a result of sitting excessively, which included bodily aches, stiffness, and inadequate blood circulation. Other participants discussed their understanding of the long-term consequences sedentary behavior could have on their functional ability and health. For example, a participant stated, “...this sedentary life would destroy me because it weakens your bones and your muscles and you can’t get up”. In addition to physical symptoms, the participants also discussed feelings of guilt, depression, boredom, and idleness after extended periods of sedentary behavior. As one participant expressed, “I agree that the thing we all should do less of is sitting down watching TV for a long time … because when you’re watching TV, you’re really not doing anything”.

Conversely, the participants shared positive attitudes toward sedentary time that afforded enjoyment, mental stimulation, and socialization. For example, one participant reported, “Doing the activities that I do—solving puzzles and at the same time talking to others at my table—helps me stay sharp”. The participants also perceived these sedentary activities as a form of rest and restoration, allowing them opportunities to relax, contemplate, and cope with environmental stressors. The participants also discussed how daily sedentary activities provided structure in their day after retirement. For example, one participant described how she began each day with sedentary time by stating: “Most of my sedentary time is listening to the radio in the morning—gospel music or some type of spiritual mediation, and then after a while, I’ll get up and move about”.

#### 3.4.2. Theme 2. Multi-Level Influences on Sedentary Behavior

The participants also discussed the personal, social, and environmental determinants of sedentary behavior within an aging context, as described below.

##### Personal and Developmental Factors

The participants shared that chronic pain and mobility limitations acted as barriers to reducing sedentary time. As one participant explained, “I have difficulty walking since I had that stroke. I used to walk a lot. Now, I paint. I work at a computer. I write…”. The participants also considered how being sedentary impacted their functional ability and continued engagement in daily activities. Many discussed their need to modify their activity patterns because of health conditions and age-related physical decline. For example, a participant stated, “I started getting arthritis in my hips and knees and also COPD. So, that’s really slowed me down. I try to walk but not that far”.

Retirement was also discussed as a key life transition that influenced current activity levels. Many saw retirement as an opportune time to engage in more of the leisurely activities that they enjoyed but did not have time for previously. As one participant revealed, “My life used to be a very routine life going to work, but now it changed because I retired. I started coming here [to the center] and began to socialize more, get to know more people, different things that I didn’t do before like exercise classes, computer classes…”. Some discussed how changes in activity level following retirement were a consequence of the type of work they performed previously. Those who held more physically demanding positions described an increase in sedentary time after retirement: “Yes, [I’ve become more sedentary] because before I worked in the field every day. So now, I don’t get up as much as I used to, and once I get up, I’m [sitting] at the TV”. Meanwhile, others, who worked more sedentary jobs, revealed how much more active they had become since retiring.

##### Interpersonal Factors

Interpersonal relationships also influenced the amount of sedentary time the participants accumulated. For instance, a participant shared how her close relationships contributed to a reduction in the time she spent sitting: “I went from being very… active to being completely out of it, to now, I’m back and I have friends now that keep me busy”. Activities that provided enjoyment and social connection were valued by the participants, regardless of whether the activities were sedentary. Activities such as puzzles and watching TV were justified if they were performed as a group and provided a shared experience. For instance, a participant stated, “We have TV discussions here. Everybody comes in and we’re all [excited] about it. I just love it! and if I missed something, somebody else could tell me what I miss. It’s fun!” Conversely, conflict with peers at the center were discussed as a stressor that potentially impacted senior center attendance, increasing the likelihood of remaining at home and engaging in sedentary behaviors.

The social climate at home or at the center was viewed as an integral part of healthy aging as it was perceived to shape the expectations of what was thought to be developmentally appropriate activities for older adults. Some participants described feeling compelled to perform certain activities seated because of prevailing social norms. The negative aging stereotypes encountered during interpersonal exchanges contributed to the participants feeling infantilized by family and friends, despite their best intentions. The participants strongly rejected the expectation that they should become more sedentary with age. As one participant remarked, “The biggest question is how to keep ourselves from getting shoved into that ‘you’re old now’; ‘it’s time to rest’; ‘you can’t’; ‘don’t push yourself too hard’ [category]”.

##### Neighborhood and Societal Factors

Inclement weather and steep or uneven terrain were cited as factors that contributed to increases in sedentary time. The availability and affordability of transportation enabled them to venture out into their local neighborhood and beyond to engage in a variety of outdoor activities. As one participant remarked, “Being in New York, it’s much easier to get around. You don’t have to spend a whole lot of money to get someplace, and there’s so many things that are available for seniors here in New York City compared to elsewhere”. The availability of and proximity to leisure opportunities locally and at the center were identified as a significant influences in discouraging sedentary behavior. As one participant reported, “That’s one thing about this place. They have so many physical things to offer us, like the one I just went to yesterday, Stretching for Arthritis, really helped!” However, others noted the resource limitations of nearby senior centers, which limited the variety and quality of the programs offered.

Neighborhood institutions, such as senior centers and places of worship, were emphasized for their critical role within the community, highlighting the importance of support and adequate funding for institutions that promote healthy aging. Therefore, the participants viewed their senior center as a key community resource that supported continued engagement in life activities and encouraged them to remain active and to flourish. The participants discussed broad societal factors as a backdrop against which healthy aging occurred by commenting on the ground-level impact of policy initiatives aimed at improving the health and wellbeing of older adults. Many acknowledged how municipal subsidies made low-cost meals and senior-center programming available. For example, one participant expressed gratitude by stating, “We have to thank the government, or the city, to have a center—a senior citizen center. They think about us having a meal, which is offered here. They offer exercise to keep us active, and I think it’s great”. Other participants thought policymakers should take greater action to ensure that older adults have the security they need to age well, as captured by a participant who observed, “If they don’t have enough food, they’ll be less healthy. They don’t have good health care, they’ll be less healthy. Well, to me, this is pretty obvious… if we need more food, then help with food stamps, and if we need better housing, then make laws that make housing more stable”.

In considering other societal determinants, some participants bemoaned how technological advancements and modern-day conveniences in contemporary society encouraged more sedentary behavior. This was captured by a participant who stated, “…Most of the things we do every day do not involve movement [to any] extreme degree at all. We don’t walk great distances. Everything is automated to the point where it leaves you with your only options for exercise is something that’s structured exercise”.

#### 3.4.3. Theme 3: Determining Own Path to Healthy Aging

Many participants maintained a positive view of aging and held higher expectations of their aging self, asserting they had ultimate control over their health, and stating that personal accountability played an important role in healthy aging. As one participant expressed, “Some of us, as we age, we cause our own deterioration because we tell ourselves, ‘Oh, I can’t do this again because I’m a certain age…”. The participants strongly believed that one’s attitude towards aging was an important contributor to healthy aging. Meanwhile, the participants who did not identify as an ‘active person’ discussed how comforting their sedentary activities were and how much it was a part of their daily routine. For others, their own perceived mortality and the limited amount of time they had left was a significant motivation for seeking comforting activities, regardless of their sedentary nature, as exemplified by a participant who declared, “At this age, I want pleasure. So, I make my choices”. The identities and life roles that developed from the participants’personal beliefs and health habits also shaped how they viewed the interaction between sedentary behavior and healthy aging. Some participants, who embraced volunteering and caregiving as part of their identity, were resolute on remaining active as they age. As one participant expressed, “I have my dog that I walk, I help take care of [an older neighbor] in my building, and I help my mom. So, between [them], I’m running back and forth…”.

These attitudes either motivated the participants to or prevented them from continuing to engage in activities despite health issues and age-related changes. Interestingly, some of the participants were critical of the term healthy aging altogether, as a participant stated, “I think [healthy aging] is a negative term in the first place. It’s like when you see the shirts, ‘Girls Can Do Anything’. Well, the only reason [they’re] wearing that is the same reason people used to wear ‘Black is Beautiful’ shirts. It’s because [they’re] trying to push back [against a prevailing norm]. ‘Healthy aging’ implies that aging is not healthy”. Despite this wrestling with terminology, the participants viewed healthy aging as being due to an accumulation of healthy habits and behaviors (i.e., staying active, proper nutrition) that were developed over a lifetime. There was also a recognition that institutional and aging policies affected the resources available to them. Many of the participants felt strongly about the need to advocate and be included in local decision-making: “I think it’s very important that we have a voice in what’s going to happen at our local center, and that it does not happen from top down”.

## 4. Discussion

The major finding of this qualitative study among community-dwelling older adults from racially, ethnically, and socioeconomically heterogeneous backgrounds was that while the participants experienced the physical and emotional distress associated with prolonged sitting, the psychological, cognitive, social, and restorative benefits derived from daily sedentary activities were valued and viewed as part of their healthy aging routine. This has important implications for the development of future programs and messaging strategies to help older adults reduce their sedentary time. There is an increasing awareness in the literature [12,45,46] of the distinct effects that different types of sedentary activities have across physical, cognitive, and psychosocial domains.

In the current study, the prevailing theme from early sedentary behavior messaging that excessive sitting is detrimental to health was consistently manifested in the focus group discussions. However, paradoxically, the participants also voiced a strong aversion to abandoning certain seated activities, as these were viewed as positive contributors to their overall health. This sample of older adults valued the enjoyable, mentally stimulating, and socially engaging seated activities in which they regularly participated. This finding adds context to the cross-sectional analysis of a national Canadian health survey conducted by O’Neill and Dogra [47], who found that cognitive and social sedentary activities were associated with better self-reported psychosocial outcomes. Similarly, Russell and Chase [48] also found that mentally or socially engaging sedentary activities may have a positive effect on the self-reported health and wellbeing of Medicare beneficiaries. In healthy aging program development, messaging should adopt a more discerning approach to sedentary behavior reduction that distinguishes sedentary activities that promote wellbeing and continue engagement from those that hinder it.

The participants with mobility limitations and gradual decline in physical function viewed seated activities, such as listening to audiobooks or socializing with peers, as positive coping strategies that they adopted to remain engaged in meaningful daily activities. This is in line with the results of a study by Chastin et al. [19], who also reported that older adults did not perceive their sitting as unhealthy, but rather as a way to manage chronic health conditions and stay functionally independent. The present research, however, builds upon previous studies through the use of a more heterogeneous sample to allow the exploration of the determinants of the sedentary behavior of community-dwelling older adults from both high- and low-income neighborhoods, as well as mixed sociocultural backgrounds.

With regard to the social and physical context, the participants were determined not to be restricted by stigmatizing aging norms, the cultural expectation that they should sit, and the paternalistic attitudes of their family members. At the same time, however, the participants asserted it was their prerogative to sit, relax, and contemplate in their late age if they so desired. Similar to findings published in the qualitative study by Palmer et al. [24], the participants in this study declared that the restorative aspect of their routine sedentary activities was a wellspring of their continued engagement. Therefore, person-centered goal setting that reflects older adults’ self-determination in their activity choices should be used to target areas within daily routines in which older adults would be most amenable to reducing their sedentary behavior, as individualized approaches ultimately benefit their self-rated health [49].

Lastly, we observed how the senior center played an essential role in helping this sample of older adults to maintain an independent lifestyle in the community. Recognized as vital community hubs where older adults can engage in healthy aging activities and avoid premature institutionalization [50], senior centers can serve as a third place [51], offering a common gathering space in which to perform social activities, engage in activities outside the home, and exercise autonomy. Frequenting the senior center likely fostered a stronger sense of belonging among the participants in this study, which may have helped them to become less sedentary [52]. These findings seem to suggest the need for public policies that support access to and availability of senior centers as a way to encourage healthy aging in the older adult population.

## 5. Strengths and Limitations

A primary strength of this study was the contextualized views on sedentary behavior and healthy aging gathered from a racially, ethnically, and socioeconomically diverse group of older adults. This research also emphasizes the importance of participant engagement when designing and implementing evidence-based programs to promote healthy aging. However, there are important limitations to consider. The use of a convenience sampling method, applied in a high-density urban location, limits the transferability to senior centers in other geographic regions and to other populations, including those that may be more sedentary or less mobile because of health issues. Moreover, the use of subjective measures to identify the activity patterns and sedentary behavior of the participants may have introduced social desirability bias. Furthermore, the sedentary behavior questionnaire used in this study combined two previously validated instruments but was not validated itself. Lastly, the study design did not include an in-depth analysis of the reported sedentary time to quantify a time point at which the participants perceived they were sitting for too long and sought to discontinue this behavior to avoid experiencing the symptoms associated with prolonged sitting. Future studies should consider engaging older adults in discussions surrounding such a sitting threshold and its influence on their health behavior.

## 6. Conclusions

This study explored the perceptions of sedentary behavior and healthy aging among a heterogeneous group of community-dwelling older adults. Although the participants experienced the physical consequences of extended periods of sitting, value was placed on the psychological, cognitive, and social benefits of the routine sedentary activities in which they engaged. With a higher prevalence of sedentary behavior observed among women and minority populations, it is important to better understand prevailing perceptions to design more acceptable programs. In sharing their insights and recommendations for senior-center programs to reduce sedentary behavior, the participants in this study were able to exercise control and self-determination in their lives—key pillars of healthy aging.

## Figures and Tables

**Table 1:** Demographic characteristics of total participants and by center ^1^.

	Total	Senior Center A	Senior Center B
	(*n* = 46)	(*n* = 22)	(*n* = 24)
Age, years	75.6 [7.8]	74.8 [9.3]	76.3 [6.4]
Gender, female	41 (89.1%)	21 (95.5%)	20 (83.3%)
Race/Ethnicity			
Black, or of Caribbean or African descent	27 (60.0%)	20 (95.2%)	7 (29.2%)
White, or of European descent	11 (24.4%)	-	11 (45.8%)
Hispanic or Latino	2 (4.4%)	-	2 (4.4%)
Other	5 (11.1%)	1 (4.8%)	4 (16.7%)
Educational attainment			
College degree or higher	22 (48.9%)	2 (9.5%)	20 (83.3%)
Some College	11 (24.4%)	8 (38.1%)	3 (12.5%)
High School Diploma/GED	9 (20.0%)	8 (38.1%)	1 (4.2%)
Less than High School	3 (6.7%)	3 (14.3%)	-
Employment Status			
Retired	39 (89.7%)	17 (77.3%)	22 (95.7%)
Disabled	4 (8.9%)	3 (13.6%)	1 (4.4%)
Employed	2 (4.4%)	2 (9.1%)	-
Relationship Status			
Single, never married	10 (22.7%)	6 (30.0%)	4 (16.7%)
Married/committed relationship	5 (11.4%)	1 (5.0%)	4 (16.7%)
Widowed	15 (34.1%)	9 (45.0%)	6 (25.0%)
Divorced	11 (25.0%)	3 (15.0%)	8 (33.3%)
Separated	3 (6.8%)	1 (5.0%)	2 (8.3%)
Living Status			
Lives alone	28 (60.9%)	15 (68.2%)	13 (54.2%)
Lives with spouse/partner	5 (10.9%)	1 (4.5%)	4 (16.7%)
Lives with other family members	8 (17.4%)	6 (27.3%)	2 (8.3%)
Other	5 (10.4%)	-	5 (20.8%)
How often do you visit the center?			
Daily	17 (37.0%)	12 (54.5%)	5 (20.8%)
A few times a week	21 (45.6%)	8 (36.4%)	13 (54.2%)
Once a week	6 (13.0%)	-	6 (25.0%)
A few times a month	2 (4.4%)	2 (9.1%)	-
How do you usually get to the center?			
Walk or bike	30 (65.2%)	21 (95.5%)	9 (37.5%)
Public transit	10 (21.5%)	-	10 (41.7%)
Para-transit	4 (8.7%)	1 (4.6%)	3 (12.5%)
Private Car	2 (4.4%)	-	2 (8.3%)

^1^ Mean (SD) or *n* (%).

**Table 2:** Self-reported health status and activity patterns of total participants ^1^.

	Total (*n* = 46)
General physical health is?	
Excellent	5 (10.87%)
Very good	16 (34.8%)
Good	20 (43.5%)
Fair/Poor	5 (10.9%)
General mental health is?	
Excellent	10 (21.7%)
Very Good	17 (36.97%)
Good	13 (28.3%)
Fair/Poor	6 (13.0%)
Compared to my peers, I am...	
More physically active	31 (68.9%)
About the same	10 (22.2%)
Less physically active	4 (8.9%)
Self-reported intensity and frequency of physical activities	
Over the past 30 days, …	
…did you walk or bike to do you errands?	
Yes	38 (82.6%)
No or unable	8 (17.4%)
…did you do any vigorous recreational activities for at least 10 min?	(*n* = 45)
Yes	24 (53.3%)
No or unable	19 (42.2%)
I don’t know	2 (4.4%)
If yes, how often?	(*n* = 24)
Once a week	4 (16.7%)
Most days a week	18 (75.0%)
Every day	1 (4.2%)
I don’t know	1 (4.2%)
…did you do any moderate-intensity recreational activities for at least 10 min?	(*n* = 44)
Yes	33 (75.0%)
No or unable	9 (20.5%)
I don’t know/unable	2 (4.5%)
If yes, how often?	(*n* = 33)
Once a week	4 (12.1%)
Most days a week	20 (60.6%)
Every day	7 (21.2%)
I don’t know	2 (6.1%)
…did you do any heavy work in the house or yard for at least 10 min?	(*n* = 44)
Yes	23 (52.3%)
No	18 (40.9%)
I don’t know	3 (6.8%)
If yes, how often?	(*n* = 23)
Once a week	5 (21.7%)
Most days a week	13 (56.5%)
Every day	2 (8.7%)
I don’t know	3 (13.0%)
Mean (SD) self-reported total sitting time	
In the past week, I typically sat for ____ hours a day	4.5 [2.0]

^1^ Mean (SD) or *n* (%).

**Table 3:** Themes, core categories, and sub-categories identified from focus group discussions.

Theme 1: Avoiding Sedentary Behavior, Enjoying Seated Activities	
Costs of sedentary behavior	Bodily aches, stiffness, poor blood circulation Loss of muscle strength and fitness Feelings of guilt, low mood, boredom, idleness
Benefits of sedentary behavior	Enjoyment, mental stimulation, and socialization Restoration (rest, contemplation) Coping with physical limitations, environmental stressors Structuring daily routines
**Theme 2. Multi-level Influences on Sedentary Behavior**	
Personal and developmental factors	Health status (mobility limitations, chronic pain, age-related physical decline) Retirement and type of job held previously
Interpersonal factors	Connections (or conflict) with family and peers Social climate and aging expectations
Neighborhood and societal factors	Physical terrain and weather Availability and affordability of transportation Acceptability of local leisurely activities Senior centers and other key community institutions
**Theme 3.** **Determining Own Path to Healthy Aging**	
Attitudes towards aging	Staying active to hinder deterioration Appreciating rest and comfort in the context of mortality Being accountable for own health Advocating for supportive aging policy and inclusion in decision-making

## Data Availability

The data presented in this study are available on request from the corresponding author.
